# Hyperoxia provokes gut dysbiosis in rats

**DOI:** 10.1186/s13054-020-03247-0

**Published:** 2020-08-24

**Authors:** Zhouxiong Xing, Yunhang Li, Guoyue Liu, Ying He, Yuanfa Tao, Miao Chen

**Affiliations:** 1 Department of Critical Care Medicine, Affiliated Hospital of Zunyi Medical University, Zunyi, 563000 Guizhou Province China; 2 Department of Cardiology, Affiliated Hospital of Zunyi Medical University, Zunyi, 563000 Guizhou Province China; 3 Department of Critical Care Medicine, The Second Affiliated Hospital of Zunyi Medical University, Zunyi, 563000 Guizhou Province China; 4 Department of Pancreatic Surgery, Wuhan University Renmin Hospital, Wuhan, 430060 Hubei Province China

**Keywords:** Oxygen therapy, Hyperoxia, Gut microbiota, Gut dysbiosis

Oxygen therapy is widely used in critically ill patients and usually exposes patients to hyperoxia, resulting in adverse clinical outcomes [1]. Many studies have explored the adverse effects of hyperoxia in the lung, heart, and brain. Gut microbiota plays an important role in human health and disease [2]. However, the impact of hyperoxia on gut microbiota remains unclear, and studies are limited and have yielded contradictory results [3, 4]. We attempted to explore the effect of hyperoxia on gut microbiota by exposing rats to normobaric oxygen for 7 days.

The experimental protocol was approved by the Institutional Animal Care and Use Committee at Zunyi Medical University. Male Sprague-Dawley rats (8 weeks of age, all the same strain) were obtained from the Kavans Laboratory Animal Company (Changzhou, China). All animals had free access to the same chow and water and were maintained in the same containers. The rats were pooled and randomly divided into the control group (*n* = 9) and oxygen group (*n* = 9). The oxygen group was exposed to 80% normobaric oxygen for 7 days in a hyperoxia chamber (Changjintech, Changsha, China). The control group was reared in another chamber with room air for 7 days. Fecal pellets were collected at days 0 and 7, and DNA was extracted and prepared for 16S ribosomal RNA V3–V4 region gene sequencing. Sequencing libraries were sequenced on an Illumina MiSeq platform at Biomarker Technologies Company (Beijing, China). Strain composition analysis and beta diversity analysis were performed. We used linear discriminant analysis (LDA) with effect size measurements for the quantitative analysis of biomarkers within different groups.

Figure 1 shows the relative bacterial abundance at the phylum level and the beta diversity analysis between the groups. At day 0, a principal coordinates analysis (PCA) plot showed that the difference between the two groups was not statistically significant, based on unweighted UniFrac distances (*R*^2^ = 0.086, *p* = 0.055) (Fig. 1b). At day 7, the PCA plot showed that the scatter points of the two groups were discrete, and the difference between the groups was statistically significant, based on unweighted UniFrac distances (*R*^2^ = 0.185, *p* = 0.001) (Fig. 1d). It was demonstrated that 80% oxygen changed the composition of the gut microbiome. Further LDA analysis showed the enriched bacteria in the two groups at day 7 (Fig. 2). Focusing on the pathogenic bacteria, we found that *Streptococcus* was enriched in the oxygen group, but Gammaproteobacteria and *Proteus* were enriched in the control group.

**Fig. 1:**
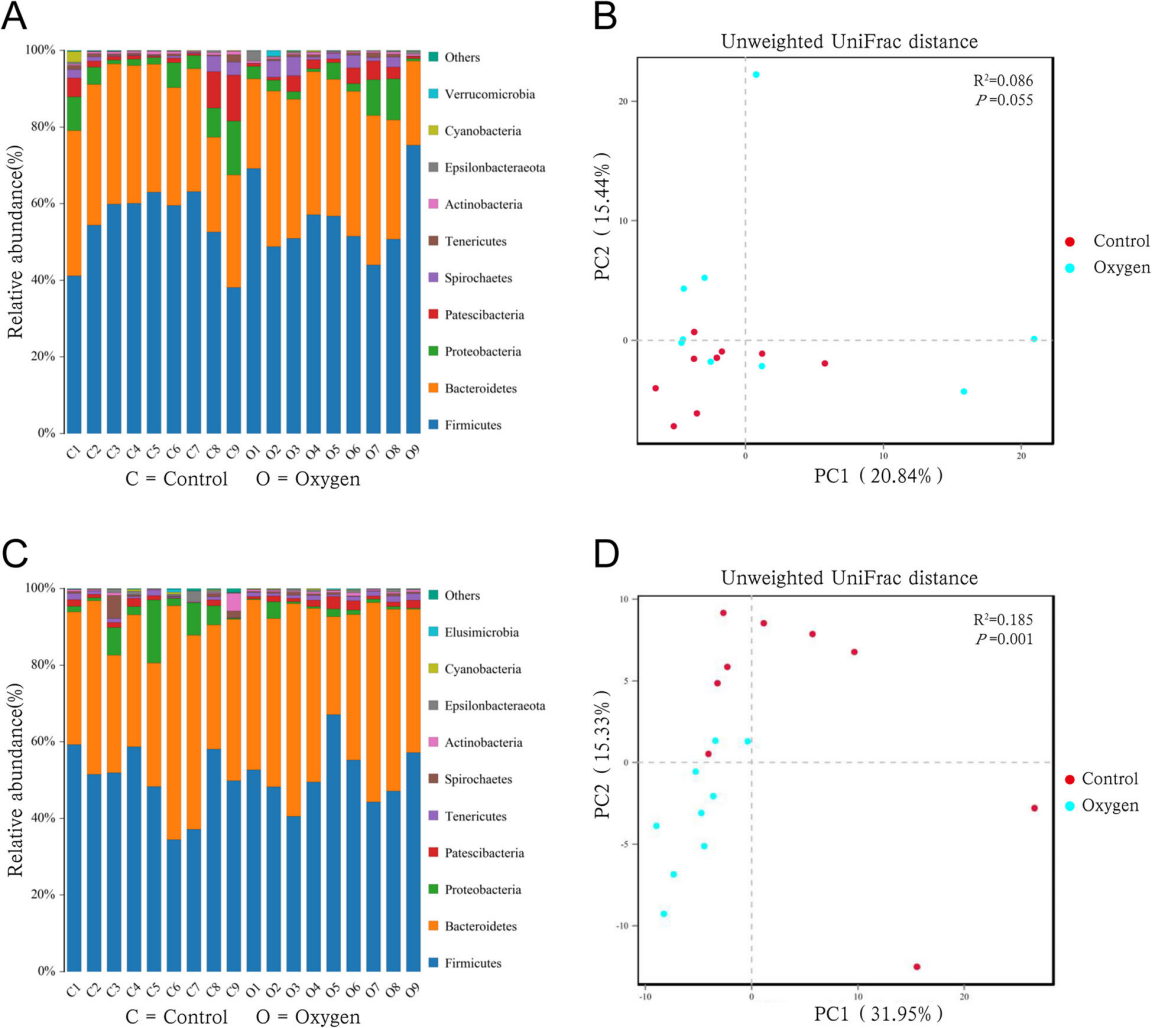
Relative bacterial abundance at the phylum level and beta diversity. **a** Relative bacterial abundance of the control and the oxygen groups (*n* = 9) at the phylum level at day 0. **b** PCA plot of the control and the oxygen groups (*n* = 9) at day 0 based on unweighted UniFrac distances (*R*^2^ = 0.086, *p* = 0.055). **c** Relative bacterial abundance of the control and the oxygen groups (*n* = 9) at the phylum level at day 7. **d** PCA plot of the control and the oxygen groups (*n* = 9) at day 7 based on unweighted UniFrac distances (*R*^2^ = 0.185, *p* = 0.001**). PCA, principal coordinates analysis. **p < 0.01. The corresponding phyla of the pathogenic bacteria in this study: Proteobacteria (Gammaproteobacteria and *Proteus*) and Firmicutes (*Streptococcus*)

**Fig. 2:**
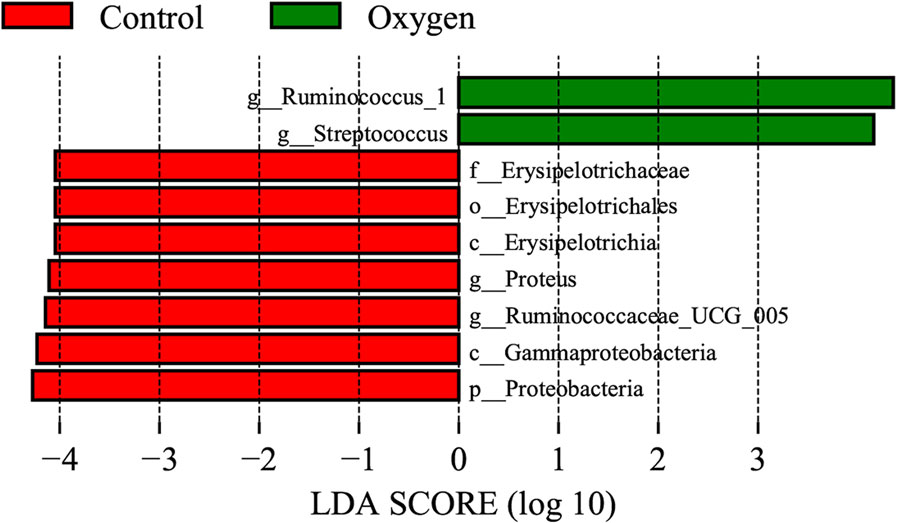
LDA along with effect size measurements was applied to the enriched bacteria from the genus level to the phylum level in the control and oxygen groups at day 7 (*n* = 9). LDA, linear discriminant analysis

To date, a great amount of work has been carried out in hyperoxia-related organ damage, basically and clinically. However, very few studies have explored the impact of hyperoxia on intestinal microbiota [5]. A previous study has indicated that hyperbaric hyperoxia alters the composition of the gut microbiota in mice, and one lineage, *Anaerostipes*, an obligately anaerobic Firmicute, diminishes after hyperbaric hyperoxia [3]. However, a recent study has suggested that normobaric hyperoxia cannot change the gut microbiota in rat pups [4]. However, this study was limited by its small sample size (*n* = 4). In our study, we found gut dysbiosis induced by normobaric hyperoxia in an adult rodent model. Our model consisted of a larger sample size. Compared to hyperbaric oxygen therapy, normobaric oxygen therapy can expose patients to oxygen for a longer time and is far more widely used in various settings [6]. It is important to know how normobaric hyperoxia influences the gut microbiota. In our study, we also found that hyperoxia influences some pathogenic bacteria, enriching *Streptococcus* and diminishing Gammaproteobacteria and *Proteus*. A possible reason for this different behavior is that hyperoxia has specific selective effects in different bacteria.

In conclusion, hyperoxia provokes gut dysbiosis in rats, in a complex manner.

## Data Availability

The authors state that all the data are true and available in the research letter.
